# Probing the Magnetic Ground State of Ba_2_YIrO_6_: Impact of Nonmagnetic Dopants and Spin–Orbit Coupling

**DOI:** 10.3390/ma17081766

**Published:** 2024-04-11

**Authors:** Shuvajit Halder, Md Salman Khan, Fabrice Bert, Payel Aich, Carlo Meneghini, Sugata Ray

**Affiliations:** 1 School of Materials Sciences, Indian Association for the Cultivation of Science, 2A & 2B Raja S. C. Mullick Road, Jadavpur, Kolkata 700032, Indiamssr@iacs.res.in (S.R.); 2 Laboratoire de Physique des Solides, UMR CNRS 8502, Universite Paris-Sud, 91405 Orsay, France; 3 Dipartimento di Scienze, University Roma Tre, Via della Vasca Navale 84, 00146 Rome, Italy; payelaich2104@gmail.com; 4 LASR3 Surface Analysis Laboratory, University Roma Tre, Via della Vasca Navale 84, 00146 Rome, Italy; 5 Technical Research Center, Indian Association for the Cultivation of Science, 2A & 2B Raja S. C. Mullick Road, Jadavpur, Kolkata 700032, India

**Keywords:** iridates, spin–orbit coupling, local-structure, *jj*-coupling, doping, magnetism

## Abstract

Strong spin–orbit coupling (SOC) in iridates has long been predicted to lead to exotic electronic and magnetic ground states. ${\mathrm{Ba}}_{2}$YIr$O_{6}$ (BYIO) has attracted particular attention due to the expectation of a $J_{\mathrm{eff}}$ = 0 state for ${\mathrm{Ir}}^{5+}$ ions under the jj-coupling scheme. However, controversies surround the actual realization of this state, as finite magnetic moments are consistently observed experimentally. We present a multi-physics study of this system by progressively introducing nonmagnetic ${\mathrm{Sb}}^{5+}$ ions in place of ${\mathrm{Ir}}^{5+}$ (${\mathrm{Ba}}_{2}$YIr${}_{1-y}$Sb${}_{y}$O${}_{6}$, BYISO). Despite similar charge and ionic radii, Sb${}^{5+}$ doping appears highly inhomogeneous, coexisting with a fraction of nearly pure BYIO regions, as confirmed by X-ray diffraction (XRD). This aligns with observations in related compounds. While inhomogeneity creates uncertainty, the doped majority phases offer valuable insights. It is relevant that the inclusion of even small amounts of Sb${}^{5+}$ (10–20%) leads to a rise in magnetization. This strengthens our previous suggestion that magnetic Ir ions form dynamic singlets in BYIO, resulting in a near-nonmagnetic background. The observed moment enhancement with nonmagnetic doping supports the breakdown of these singlets. Furthermore, the magnetization steadily increases with an increasing Sb${}^{5+}$ content, contradicting the anticipated approach towards the $J_{\mathrm{eff}}$ = 0 state with increased SOC due to reduced hopping between Ir${}^{5+}$ ions. This reinforces the presence of individual Ir${}^{5+}$ moments. Overall, our findings suggest that Ba${}_{2}$YIrO${}_{6}$ might not possess sufficiently strong SOC to be solely described within the jj-coupling picture, paving the way for further investigation.

## 1. Introduction

Exotic electronic and magnetic ground states due to the influence of strong spin–orbit coupling have been the subject of intensive study for more than a decade now. As the spin–orbit coupling is proportional to the lower power of the atomic number (proportional to $Z^{2}$–$Z^{4}$), the 4*d* and 5*d* transition metals have become the central focus of study, especially the 5*d* iridium-based compounds [1,2,3,4,5]. The interplay between the strong spin–orbit coupling (SOC) and comparable onsite Coulomb interaction (*U*), along with the crystal field effect ($\Delta_{CFE}$), inter-site hopping ($t_{ij}$), Hund’s coupling ($J_{H}$), and superexchange interaction energy 4$t^{2}$/*U*, drives the system into many rich quantum mechanical states [3,6], such as Mott insulators [7], Weyl semimetals [8], quantum spin liquids [9,10], topological insulators [3,8], etc. In the strong spin–orbit coupling regime, $m_{j}$ becomes the only good quantum number instead of $m_{l}$ (orbital) and $m_{s}$ (spin), where the total angular momentum *J* determines the multiplates and degeneracy in the system.

The study of iridates increased manifold after the reports of an SOC-driven Mott insulating ground state in layered Sr${}_{2}$IrO${}_{4}$ [6] and Na${}_{2}$IrO${}_{3}$ [11], which contradicts the uncorrelated band metallicity in iridates.

In the single-particle picture, large SOC splits the six-fold degenerate $t_{2g}$ orbitals of tetravalent iridium (Ir${}^{4+}$, 5$d^{5}$ with one hole) into four-fold degenerate fully filled $J_{\mathrm{eff}}$ = 3/2 and doubly degenerate half-filled $J_{\mathrm{eff}}$ = 1/2 states. The $J_{\mathrm{eff}}$ = 1/2 band undergoes a Mott transition due to a relatively small (compared to the bandwidth) on-site Coulomb repulsion *U* [2,3,6,12,13]. This contrasts with pentavalent iridates (Ir${}^{5+}$, 5$d^{4}$ with two $t_{2g}$ holes), where SOC leads to various (15) states, with a $J_{\mathrm{eff}}$ = 0 nonmagnetic state as the ground state. Surprisingly, to date, such an unusual nonmagnetic state with two unpaired electrons in the $t_{2g}$ band has never been realized in any kind of Ir${}^{5+}$ compounds [10,14,15,16,17,18,19]. Such deviations are often explained using different existing solid-state effects, such as the non-cubic crystal field ($\Delta_{CEF}^{NC}$), which modifies the effective SOC [11,14,20,21], the ligand–metal charge transfer, inter-site hopping, or ionic disorder [22,23], which can modify the SOC description at the atomic level and introduce small magnetic moments [7,10,14,17,19,24,25,26]. Particularly, the proposal of condensation of Van Vleck excitons, when the SOC strength and the superexchange energy scale become comparable, has been widely accepted as a feasible mechanism for moment development [24].

**Figure 1:**
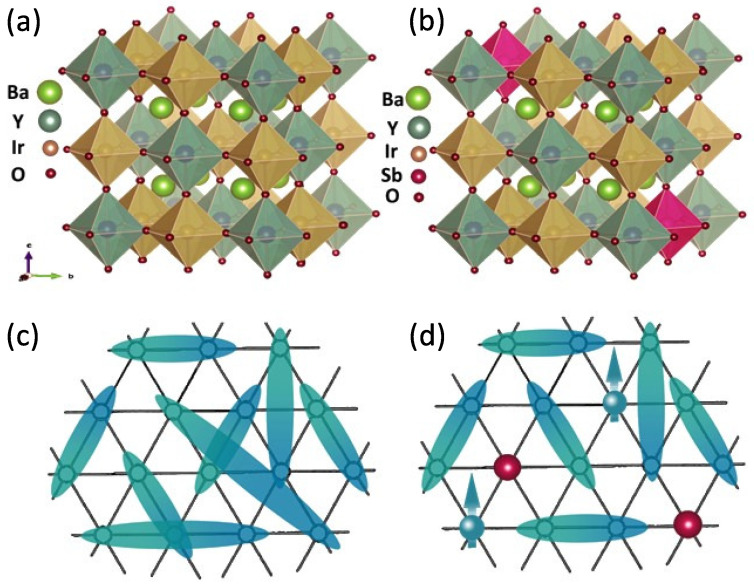
Crystal structures of (**a**) BYIO and (**b**) Sb-doped BYISO, showcasing the disruption caused by Sb doping (colour code: Green ball: Barium, Deep Green: Yttrium, Brown: Iridium, Deep Pink: Antimony, and Maroon: Oxygen). Schematic diagrams of (**c**) resonating valence bond (RVB) singlets (Cyan and Dark Cyan colour gradient) in BYIO and (**d**) antiferromagnetic ${\mathrm{Ir}}^{5+}$ (Cyan ball) singlets disrupted by nonmagnetic Sb dopants (Red ball).

In this context, the perfectly cubic perovskite Ba${}_{2}$YIrO${}_{6}$ (BYIO) containing Ir${}^{5+}$ generated quite a bit of curiosity. Surprisingly, despite being free of any non-cubic crystal field ($\Delta_{CEF}^{NC}$ = 0) effect, various studies have consistently reported finite magnetic moments for BYIO ranging from 0.16 $\mu_{B}$/Ir to 1.44 $\mu_{B}$/Ir with no long-range magnetic order observed down to the lowest measured temperature of 60 mK [17,19,26,27,28]. However, the origin of these observed magnetic moments is a subject of intense debate. A significant body of research, encompassing both experimental and theoretical investigations, has claimed that BYIO is truly a nonmagnetic $J_{\mathrm{eff}}$ = 0 system. These researchers attribute the observed magnetization solely to sample-related issues [1,22,23,27,29,30,31,32]. Contrarily, others argue that BYIO is intrinsically magnetic like other iridates, with each Ir${}^{5+}$ ion retaining a finite moment. [17,26,28,33,34,35]. Our group previously concluded the existence of hopping-induced finite intrinsic Ir moments in BYIO, forming fluctuating nonmagnetic Ir-Ir singlets with no long-range order (Figure 1a), resulting in a net nonmagnetic state between 60 mK and 10 K, as confirmed by μ-SR measurements [19]. In order to validate this hypothesis, earlier we attempted to weaken inter-site hopping and potentially drive the system towards the predicted atomic SOC-driven nonmagnetic $J_{\mathrm{eff}}$ = 0 ground state, by diluting Ir${}^{5+}$ ions in BaYSbO${}_{6}$ (Ba${}_{2}$YIr${}_{1-y}$Sb${}_{y}$O${}_{6}$ with *y* = 0.7–0.9), which has the same cubic structure as BYIO, but instead of Ir${}^{5+}$ there is Sb${}^{5+}$, which is not magnetic [16] In this work we denote the content of Sb in BYIO by *y*, being complementary to the previous study [16], which focused on the dilution of Ir ions into a nonmagnetic structure, in which we denoted the content of Ir by *x* = 1 − *y*). However, this previous study [16] revealed an unexpected trend: the magnetic moments, instead of decreasing, exhibited a systematic increase with an increasing Sb${}^{5+}$ concentration (*y*). This unanticipated result raises concerns about the true strength of Ir${}^{5+}$ SOC in BYIO and necessitates a deeper understanding.

In the present study, we probe the low Sb doping regime, i.e., *y* = 0.1, 0.2, and 0.5, with an aim to progressively disrupt the nonmagnetic singlet pairs [19] in Ba${}_{2}$YIrO${}_{6}$ (BYIO) (Figure 1b), in order to confirm their existence. Clearly, the inclusion of nonmagnetic Sb${}^{5+}$ in BYIO is expected to break proportionate numbers of Ir${}^{5+}$-Ir${}^{5+}$ antiferromagnetically coupled singlet pairs, leaving an increasing number of unpaired Ir${}^{5+}$ ions around the dopant Sb${}^{5+}$ and consequently increasing the net magnetization by the free Ir${}^{5+}$ moments, if at all. For the sake of completeness, we have also included a few experimental results of our earlier reported *y* = 0.8 compound (x=1−y = 0.2 in ref. [16]) in the present manuscript. Consistent with our previous observation [16], the partial immiscibility problem of Sb${}^{5+}$ within the BYIO matrix has been observed here too, especially when the doping is taken above 10%. However, even then we actually obtain a major Ba${}_{2}$YIr${}_{1-y^{\prime }}$Sb${}_{y^{\prime }}$O${}_{6}$ phase with *y* < $y^{\prime }$, which can still be utilized for the stated purpose. Consequently, we have carried out detailed structural, electronic, and magnetic studies on the series of compounds, and our detailed experiments reveal that the effective paramagnetic moments continuously increase with the addition of nonmagnetic Sb${}^{5+}$ ions in the system (from ∼0.4 $\mu_{B}$/Ir to ∼0.7 $\mu_{B}$/Ir), with $\Theta_{\mathrm{CW}}$ being always negative [16], and, here again, similar to the parent Ba${}_{2}$YIrO${}_{6}$ and high Sb-doped end systems [16], no long-range magnetic order develops. Valence band photoemission spectroscopic data established that the bandwidth of hybridized Ir 5*d*-O 2*p* decreases with increased Sb doping, as expected, and the consequent increase in magnetic moments only points towards the applicability of the localized moment LS coupling model instead of the proposed jj-coupling interaction.

## 2. Experimental Details

Poly crystalline samples of the Ba${}_{2}$YIr${}_{1-y}$Sb${}_{y}$O${}_{6}$ with doping *y* = 0.1, 0.2, 0.5, and 0.8 (abbreviated as BYISO-10, BYISO-20, BYISO-50, and BYISO-80, respectively) were synthesized by the conventional solid-state reaction technique. Stoichiometric amounts of high purity (>99%, Sigma-Aldrich, St. Louis, MO, USA) BaCO${}_{3}$, Y${}_{2}$O${}_{3}$, IrO${}_{2}$, and Sb${}_{2}$O${}_{5}$ powders were thoroughly grounded in an agate mortar. Initially, the mixture was calcined at 1173 K for 12 h to decompose carbonates. The mixture was then pressed into pellets and annealed in air at 1623 K for 72 h with a few intermittent grindings. The structural characterization and phase purity of all the compounds were checked in a Rigaku SmartLab X-ray Diffractometer (Tokyo, Japan) with a Cu $K_{\alpha }$ (λ = 1.5406 Å) X-ray source at room temperature (300 K). The powder X-ray diffraction (PXRD) data were analyzed to extract the structural information through Rietveld refinement using the Fullprof software [36,37]. The X-ray photoemission spectroscopy (XPS) experiments were carried out in an OMICRON electron spectrometer (Taunusstein, Germany), equipped with a SCIENTA OMICRON SPHERA analyzer and an Al $K_{\alpha }$ monochromatic X-ray source with an energy resolution of 0.5 eV. The in situ argon sputtering was used to clean the surface of the pellets. The Ir $L_{3}$ edge (11,215 eV) X-ray absorption fine structure (XAFS) experiments were performed at the XAFS beamline of an Elettra synchrotron radiation facility in Italy [38]. The Si(111) double crystal was used to scan the X-ray beam energy across the Ir $L_{3}$ edge. The absorption spectra were measured at room temperature in transmission geometry, using two gas-filled ionization chambers to measure incident and transmitted X-ray fluxes. The BYISO samples were grounded, mixed with boron nitride (BN) matrix in an approximately 1/10 weight ratio, pressed in thin solid pellets, and mounted on the beamline measurement chamber. The absorption edge discontinuity was approximately 0.5 for all the samples. The absorption spectra from a pure Ir foil placed after the second ionization chamber were measured at the same time and used to precisely monitor the X-ray beam energy calibration. The extended X-ray absorption fine structure (EXAFS) data were analyzed quantitatively using the open-source DEMETER (Athena and Artemis) [39,40] and ESTRA-FitEXA [41] software packages. The magnetization measurements in the temperature range of 2–300 K and ±5 T were performed in a superconducting quantum interference device (SQUID) magnetometer (Quantum Design). The muon spin resonance (μ-SR) experiments were performed using the muon spectrometer at the ISIS Neutron and Muon source facility in the United Kingdom.

## 3. Results and Discussions

### 3.1. Crystal Structure from X-ray Diffraction

**Figure 2:**
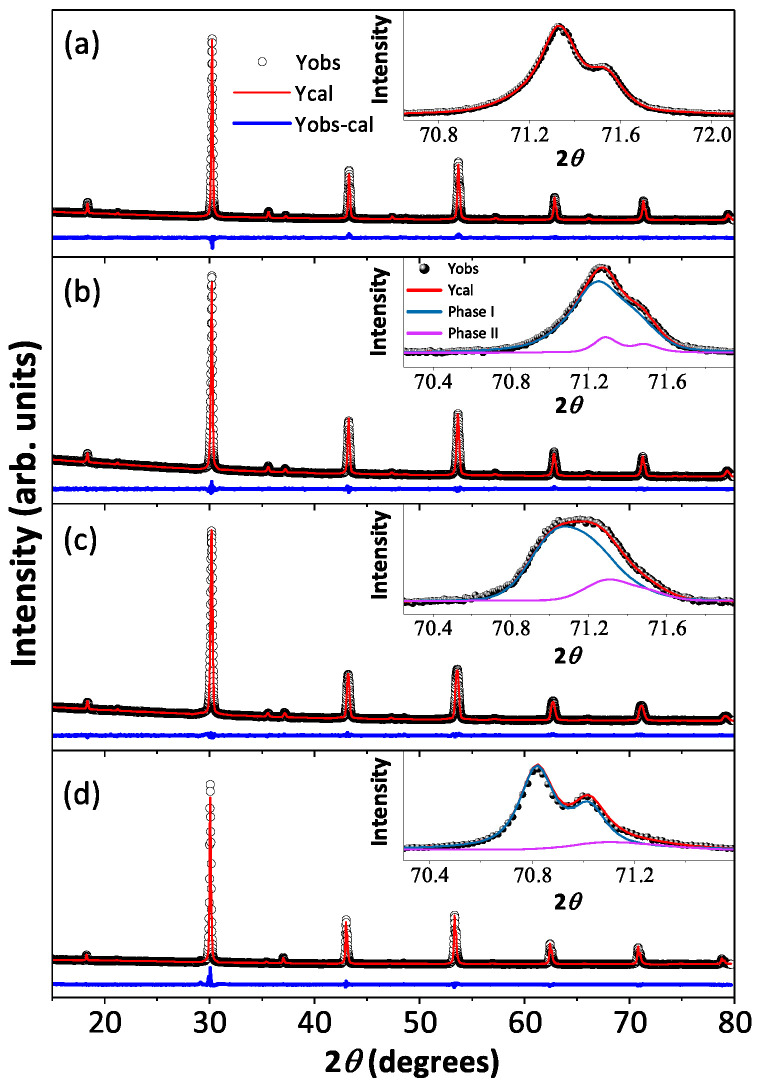
Room temperature X-ray diffraction (black open circle) along with Rietveld refined patterns (red solid line) of Ba_2_YIr${}_{1-y}$Sb${}_{y}$O${}_{6}$ are shown in (**a**) BYISO-10, (**b**) BYISO-20, (**c**) BYISO-50, and (**d**) BYISO-80 panels, respectively. The expanded view of the higher angle contribution of hkl (620) of two phases (phase I: green line; phase II: pink line) is shown in the inset of corresponding panels.

Room temperature X-ray diffraction patterns and the best-fitted Rietveld analysis curves for all the polycrystalline Ba${}_{2}$YIr${}_{1-y}$Sb${}_{y}$O${}_{6}$ (*y* = 0.1, 0.2, 0.5, and 0.8) compounds are shown in Figure 2a–d along with the high 2θ angle peak (inset to Figure 2a–d), which signify the influence of Sb on crystallographic phases as a function of doping concentrations, and the refined crystallographic lattice parameters and the phase percentages are listed in Table 1. The structural analysis reveals that the BYISO-10 has a single cubic phase, the space group is $Fm\bar{3}m$, and as the doping percentage of Sb increases, the signature of two phases becomes more prominent (see Figure 2). Above 10% Sb doping, a dominating phase Ba${}_{2}$YIr${}_{1-y^{\prime }}$Sb${}_{y^{\prime }}$O${}_{6}$ (*y* < $y^{\prime }$, phase I) and a minor phase Ba${}_{2}$YIrO${}_{6}$ (phase II), which both have the cubic space group $Fm\bar{3}m$ [42], have been found to coexist in all the compounds. As the slightly large cation Sb${}^{5+}$ ($<r{>}_{{\mathrm{Sb}}^{5+}}$ = 0.60 Å) is doped in place of Ir${}^{5+}$ ($<r{>}_{{\mathrm{Ir}}^{5+}}$ = 0.57 Å), the cubic lattice constants are increased accordingly [43] (Table 1). The reason behind this inhomogeneous replacement of Ir${}^{5+}$ by Sb${}^{5+}$ is not clear. Given the fact that Y${}^{3+}$ is substantially larger ($<r{>}_{Y^{3+}}$ = 0.90 Å) than Ir${}^{5+}$/Sb${}^{5+}$, along with charge differences (see Table 2), *B*/$B^{\prime }$ anti-site disorder is found to be only marginal in both the phases. However, the nature of Sb${}^{5+}$-O-Y${}^{3+}$-O-Sb${}^{5+}$ bonding and Ir${}^{5+}$-O-Y${}^{3+}$-O-Ir${}^{5+}$ bonding may differ because of differences in *p*-block Sb${}^{5+}$ and Ir${}^{5+}$*d*-block ions and could create certain local preferences giving rise to this partial immiscibility.

### 3.2. Local Structure from Extended X-ray Absorption Fine Structure (EXAFS)

The Ir $L_{3}$ edge EXAFS data analysis has been carried out with the aim of revealing the details of the local coordination chemistry around the average Ir absorber and, in particular, the mid-range chemical order details [45], which is mandatory to shed light on the Ir-*X* (*X* = Ir, Y, Sb) correlations. To this aim, the quantitative analysis has been carried out by applying a multi-shell refinement procedure [46] to reproduce the main structural features around the average Ir absorber in the samples till around 4.5 Å. The EXAFS data analysis necessitates the careful identification of statistically relevant and physically meaning structural signals. This task involves a trial-and-error process to establish meaningful constraints on fitting parameters, thereby minimizing correlations and improving the result reliability. Following the approach outlined in Ref. [16], we utilized local atomic clusters around Ir ions (derived from XRD data) to identify key single and multiple scattering contributions. These were then employed to calculate the photoelectron amplitude and scattering functions needed for theoretical EXAFS simulations [41].

**Figure 3:**
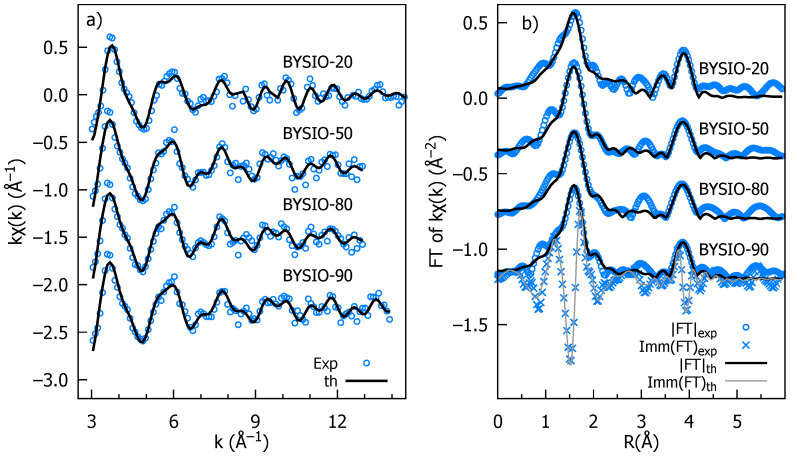
Stacked plot of Ir $L_{3}$ edge EXAFS experimental and analysis data of BYISO-10, BYISO-20, BYISO-50, and BYISO-80; each graph is labeled accordingly: (**a**) *k*-weighted experimental data and the corresponding fits in the *k* range 3–12 Å${}^{-1}$; (**b**) the Fourier transforms (moduli) of *k*-weighted EXAFS experimental data, magnitudes (FT), imaginary parts (Imm), and fitted curves.

The *k*-weighted EXAFS spectra for all the investigated samples are presented in Figure 3a, along with the best fit curves for the sake of comparison. The moduli of the Fourier transforms (|FTs|) of data and best fits are presented in Figure 3b, providing a more intuitive description of the average local atomic structure around Ir, which is a pseudo-radial distribution function in which peaks represent the average interatomic distances (or photoelectron half path length for MS terms), which are roughly 0.5 Å squeezed by the phase shift effect. The most intense peak around 2 Å is the signature of the first Ir coordination shell, consisting of the six surrounding oxygen atoms (IrO${}_{6}$ octahedra) with a coordination number (N_Ir-O_) of 6. The very equal peak intensity and shape across all four doped samples suggest a highly similar Ir-O environment with minimal distortions in the IrO${}_{6}$ octahedra, consistent with the XRD data analysis. The next evident peak in the |FT| at around 4 Å originates from the Ir next-nearest neighbors located along the perovskite cube edges. Notably, the multiple scattering (MS) contributions to this peak are significantly enhanced by the aligned Ir-O-X configurations. We accounted for Y/Ir chemical disorder, including the Ir and Y contributions whose multiplicities were by *x* and 1 − *x*, respectively, and *x* was refined. It should be noted that for all samples the best fit has *x* = 0, as expected for the ideal double perovskite, establishing the high degree of chemical order. Only the analysis of BYISO-80 reveals a fraction of antisite defects x=0.09±0.02, corresponding to averagely 5.5 Ir-Y and 0.5 Ir-Y next neighbors. This signifies that a marginal amount of antisite disorder between Ir and Y occurs only for the higher percentage of Sb doping (Table 3). We have also checked the possibility of Sb/Y antisite defects, but any attempt to include Ir-O-Sb contributions degrades the best fit quality, also in the the highest doped sample BYISO-80. The accuracy of EXAFS data analysis diminishes for shells further away either because structural disorder and the finite mean free path of the photoelectron attenuate the XAFS structural signals or because of the increasing number of interfering contributions, which lead to a complex overlap of signals from various atomic arrangements. Therefore, it is hard to comment specifically on eventual Sb substitution at the Ir position, which is around 6 Å (i.e., along the perovskite cube diagonal).

### 3.3. Oxidation State and Valence Band Spectra from X-ray Photoelectron Spectroscopy

**Figure 4:**
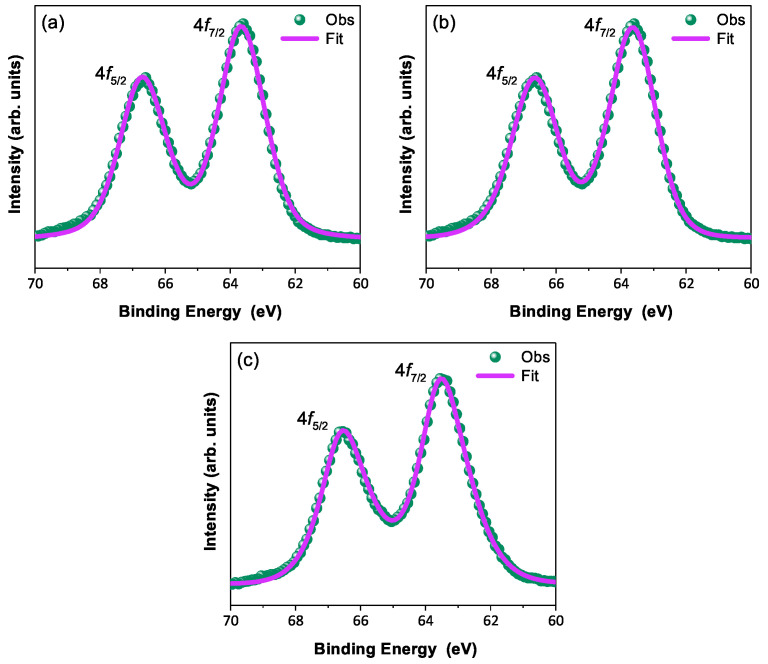
X-ray photoelectron spectroscopic spectra of 4*f* core level (green circle) with corresponding fitting (pink solid line): (**a**) BYISO-10, (**b**) BYISO-20, and (**c**) BYISO-50, respectively.

**Figure 5:**
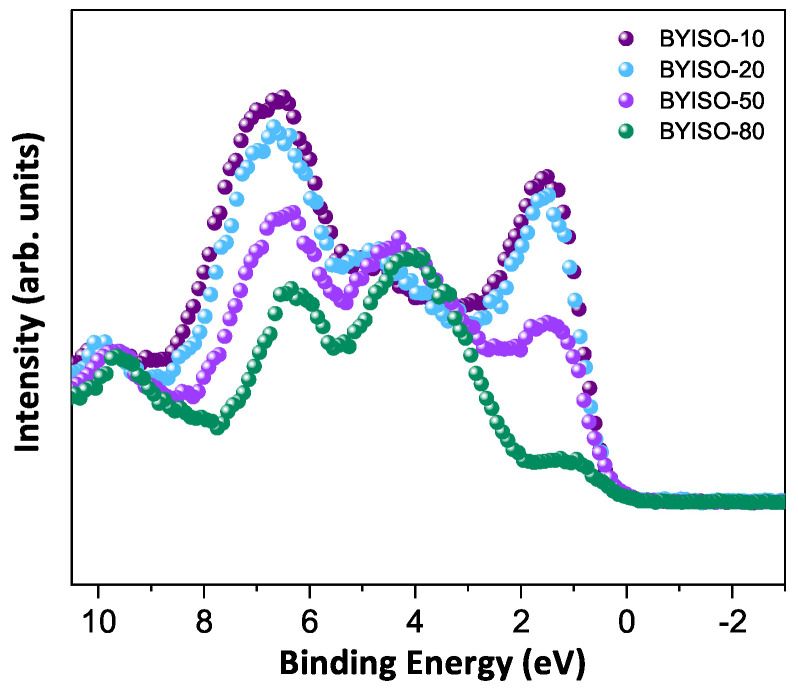
Valence band spectra of BYISO-10 (violet circle), BYISO-20 (arctic circle), BYISO-50 (pink circle), and BYISO-80 (green circle), respectively.

In order to discuss the predicted nonmagnetic ($J_{\mathrm{eff}}$ = 0) state in Ir${}^{5+}$ ions under the influence of SOC (jj-coupling), it is important to confirm the oxidation state first [6,47,48]. X-ray photoelectron spectra of the Ir 4*f* core level from all three compounds could be fitted using a single spin–orbit doublet, as shown in Figure 4. The energy positions of 4$f_{7/2}$ (63.18 eV, 63.21 eV, 63.15 eV, and 63.13 eV) and 4$f_{5/2}$ (66.23 eV, 66.25 eV, 66.19 eV, and 66.17 eV) and their spin–orbit separations of around 3.05 eV, 3.04 eV, 3.04 eV, and 3.04 eV (listed in Table 4) for BYISO-10, BYISO-20, BYISO-50, and BYISO-80, respectively, confirm the presence of pure Ir${}^{5+}$ only in all the compounds. The valence band (VB) spectra of these four compounds (shown in Figure 5) shows the absence of the density of states (DOS) at the Fermi level, indicating the insulating behavior in all of them that is exactly like the end members, Ba${}_{2}$YIrO${}_{6}$ and Ba${}_{2}$YSbO${}_{6}$. It is clearly seen from the VB spectra that as the Sb doping increases there is a gradual decrease in the DOS near to the Fermi level, indicating that this DOS is contributed mostly by the Ir 5*d*-band, and such a depletion makes the system more insulating, similar to Ba${}_{2}$YSbO${}_{6}$, which is a highly insulating dielectric compound [19,44,49].

### 3.4. Magnetic Susceptibility

**Figure 6:**
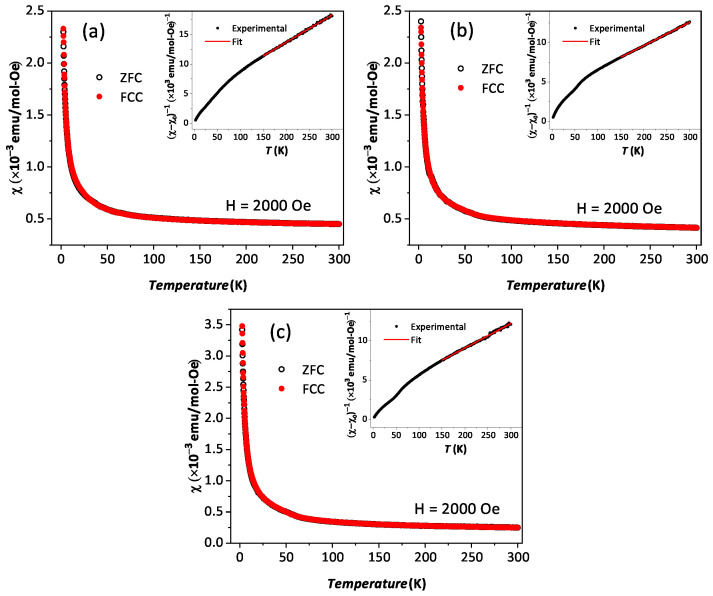
Magnetic susceptibility χ vs. T in zero field-cooled (black open circle) and field-cooled (red solid circle) contexts along with corresponding 1/(χ−$\chi_{0}$) vs. T in the inset are plotted with Curie–Weiss fitting (red solid line): (**a**) BYISO-10, (**b**) BYISO-20, and (**c**) BYISO-50.

**Figure 7:**
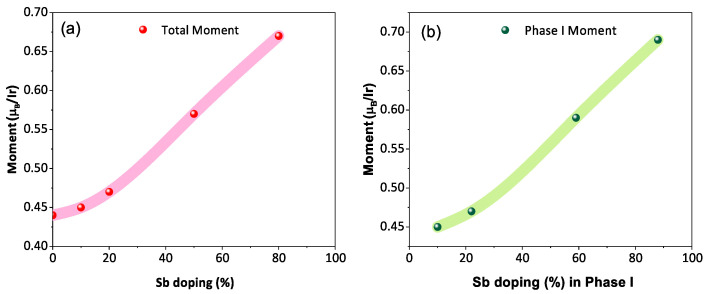
(**a**) Net magnetic moment of Ba${}_{2}$YIr${}_{1-y}$Sb${}_{y}$O${}_{6}$ with respect to the stoichiometric Sb doping percentage; (**b**) magnetic moment of phase I as a function of the effective Sb doping percentage in phase I.

The temperature-dependent (2 K to 300 K) dc magnetic susceptibility of BYISO-10, BYISO-20, BYISO-50, and BYISO-80 has been measured in zero field-cooled (ZFC) and field-cooled (FC) protocols with a 2000 Oe magnetic field, as shown in Figure 6a–c, respectively. The χ vs. *T* curves were fitted at the higher-temperature region using Curie–Weiss (CW) law: $\chi =\chi_{0}+\frac{C}{T-\Theta_{\mathrm{CW}}}$, where $\chi_{0}$, *C*, and $\Theta_{\mathrm{CW}}$ represent the temperature-independent paramagnetic susceptibility, Curie constant, and Curie–Weiss temperature, respectively [50]. The linear fittings 1/(χ − $\chi_{0}$) are satisfactory down to 150 K for BYISO-10, BYISO-20, and BYISO-50, below which they deviate from the paramagnetic behavior (inset Figure 6a–c). The dc magnetic susceptibility curves for all the compounds show no long-range ordering down to 2 K, and the $\Theta_{\mathrm{CW}}$ values are largely negative, hence the frustration parameter *f* = $\left|\Theta_{\mathrm{CW}}\right|$/$T_{N}$ values are high. The negative $\Theta_{\mathrm{CW}}$ values also indicate antiferromagnetic interaction between the Ir${}^{5+}$ ions in all the samples. However, contrary to the expectations, the net effective magnetic moments extracted from the CW fitting (listed in Table 5) are found to increase gradually with an increasing nonmagnetic Sb content, and the most isolated Ir${}^{5+}$-ion (in BYISO-80) possesses the highest moment (Figure 7a) [16]. This observation clearly indicates the following: (a) each Ir${}^{5+}$ ion possesses a finite moment, and, as a result of breaking the singlets with Sb doping, the Ir free spins and net magnetization increase; (b) increasing the Ir dilution expectedly narrows down the Ir bandwidth, but instead of moving towards the atomic $J_{\mathrm{eff}}$ = 0 limit, it takes the system more and more away from it, further confirming the fact that the system is still better described by a moderate SOC picture.

Effective paramagnetic moments of phase I (listed in Table 5) are calculated using the phase percentage observed from the XRD analysis (Table 1) and considering the effective paramagnetic moment of BYIO, i.e., phase II as 0.44 $\mu_{B}$/Ir [17].

### 3.5. Muon Spin Relaxation—μSR

**Figure 8:**
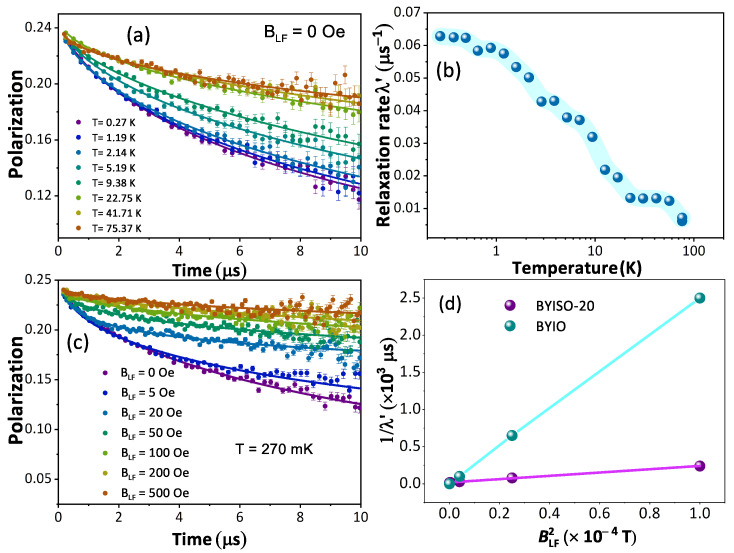
(**a**) Time evolution of the muon polarization of Ba${}_{2}$YIr${}_{0.8}$Sb${}_{0.2}$O${}_{6}$ in zero fields with fits to a stretched exponential function (continuous lines) with temperature variation; (**b**) fitted relaxation parameter $\lambda^{\prime }$ as a function of temperature (*T*); (**c**) time evolution of the muon polarization with different applied longitudinal fields with fits to a stretched exponential function (continuous lines) at T = 270 mK; (**d**) 1/$\lambda^{\prime }$ vs. $B_{LF}^{2}$ for BYIO [19] (cyan color) and BYISO-20 (magenta color) data extracted using Redfield formula (Equation (1)).

From the macroscopic magnetic measurements, such as dc magnetic susceptibility measurement, it is hard to understand the complex magnetic ground state of these cubic double perovskite systems. Hence, to understand the magnetic nature of the BYISO system it becomes important to perform a μSR experiment, which is highly sensitive to the tiny internal magnetic field due to the large gyromagnetic ratio of the muon ($\gamma_{\mu }$ = 851.615 MHz/T). Polarization P(t) variations with time at different temperatures under a zero applied field from BYISO-20 are shown in Figure 8a. It is observed that till the lowest measured temperature of 270 mK, the system is not magnetically ordered, almost similar to Ba${}_{2}$YIrO${}_{6}$ [19]. Under the application of zero external fields, the polarization at different temperatures follows the stretched exponential function, P(t) = $e^{-{\left(\lambda^{\prime }t\right)}^{\beta }}$. Fitted values of the relaxation rate (λ${}^{\prime }$) and stretched exponent (β) as a function of temperature are plotted in Figure 8b and are almost similar to the parent compound Ba${}_{2}$YIrO${}_{6}$ [19]. As the temperature decreases, the λ${}^{\prime }$ increases gradually, and below 1 K it becomes constant till 270 mK. The characteristic spin fluctuation frequency ν, defined as ν ∝ 1/λ${}^{\prime }$, becomes nearly constant below 1 K till 270 mK. The spin dynamics signifies no magnetic freezing until the base temperature (270 mK). To explore the origin of the relaxation at low temperatures, the longitudinal field (B${}_{LF}$)-dependent polarization has been measured at 270 mK. A significant variation in relaxation is observed with changes in the external longitudinal field $B_{LF}$ (shown in Figure 8c). To estimate the local field ($B_{\mu }$) that the muons experience and the fluctuation frequency (ν), the Redfield formula (Equation (1)) is used to fit the relaxation (λ${}^{\prime }$) as a function of the external applied longitudinal field B${}_{LF}$ (see Figure 8d, plotted 1/λ${}^{\prime }$ as a function of $B_{LF}^{2}$).

(1) $$\lambda^{\prime }=\lambda_{0}^{\prime }+\frac{\nu \gamma_{\mu }^{2}B_{\mu }^{2}}{\left(\nu^{2}+\gamma_{\mu }^{2}B_{LF}^{2}\right)}$$

The outcome from the Redfield formula yields $B_{\mu }$ ∼ 2.2 mT and ν ∼ 0.1 MHz, which strongly support the magnetization measurement data that 20% Sb doping in BYIO increases the internal magnetic field almost 10 times higher compared to the parent compound BYIO with less fluctuation [19]. Hence, it is clear that some resonating valence bond singlets are broken due to doping, giving rise to free Ir${}^{5+}$ spins, which contribute to this enhanced local magnetic moment [51].

## 4. Conclusions

We conducted a comprehensive multi-physical characterization of Sb-doped Ba${}_{2}$YIr${}_{1-y}$Sb${}_{y}$O${}_{6}$ (BYISO) samples to investigate the nonmagnetic Ir${}^{5+}$-Ir${}^{5+}$ singlets and shed light on the magnetic state of isolated Ir${}^{5+}$. Our structural investigations revealed the presence of defects, which are challenging to eliminate entirely in any real samples. Notably, XRD analysis showed a weak residual presence (around 10%) of a secondary BYIO phase (phase II). This results in a slight difference in the nominal composition of the Sb-doped phase (phase I), but such a difference does not exhibit a monotonic trend with the doping concentration that may motivate the evolution of magnetic properties (see below). The Ir-L${}_{3}$ edge XAFS analysis did not reveal any defects in the Ir coordination (IrO${}_{6}$ octahedra) or the presence of Ir-Sb correlations from Sb/Y substitutional defects, even at the highest Sb concentrations. A weak fraction (<10%) of Ir/Y antisite defects providing some Ir-O-Ir units was observed only at the highest Sb doping (BYISO-80).

The magnetic properties exhibit a clear progressive increase in the total sample magnetization, as well as the magnetization attributed solely to phase I (Sb-doped). Notably, the magnetization is highest in the sample with the highest Sb concentration (BYISO-80), where the Ir ions should be completely isolated. This finding clearly indicates that each Ir${}^{5+}$ ion possesses an intrinsic magnetic moment. Breaking the singlets with Sb doping leads to an increase in free Ir spins and so in the net magnetization. As expected, increasing the Ir dilution narrows the Ir bandwidth. However, contrary to the expectation of approaching the atomic $J_{\mathrm{eff}}$ = 0 limit, this effect pushes the system further away from it, reinforcing the idea that the system is better described by a moderate SOC picture. The μSR measurements further support this hypothesis. They show an order of magnitude increase in the internal magnetization field for the BYISO-20 sample compared to the pure BYIO sample. This effect evidently arises from the free Ir${}^{5+}$ spins originating from the Sb broken singlets, which enhance the local magnetic moments, against the $J_{\mathrm{eff}}$ = 0 ground state model,

Overall our results clearly demonstrate the inadequacy of a $J_{\mathrm{eff}}$ = 0 state for Ir${}^{5+}$ and suggest that the SOC strength in BYIO is better described within the framework of LS coupling.

## Figures and Tables

**Table 1:** Room temperature crystallographic information of Ba${}_{2}$YIr${}_{1-y}$Sb${}_{y}$O${}_{6}$ (*y* = 0.1, 0.2, 0.5, and 0.8): two cubic phases of space group $Fm\bar{3}m$ (space group no. 225, *a* = *b* = *c*, α = β = γ = 90${}^{\circ }$) are used to refine the powder XRD patterns. BYISO-10: R${}_{p}$ = 10.4, R${}_{wp}$ = 7.8, R${}_{exp}$ = 4.76, $\chi^{2}$ = 2.68; BYISO-20: R${}_{p}$ = 11.1, R${}_{wp}$ = 8.08, R${}_{exp}$ = 5.9, $\chi^{2}$ = 1.87. BYISO-50: R${}_{p}$ = 13.1, R${}_{wp}$ = 8.33, R${}_{exp}$ = 6.81, $\chi^{2}$ = 1.49. BYISO-80: R${}_{p}$ = 19.6, R${}_{wp}$ = 16.3, R${}_{exp}$ = 7.19, $\chi^{2}$ = 5.14 [16]. Standard uncertainty on the last digit of refined parameters is reported in parentheses. Values of the fixed or constrained parameters have no uncertainty.

Sample	Phase (%)	a (Å)	Atom	Occupancy	x	y	z	B (Å)${}^{2}$
BYISO-10	Ba${}_{2}$YIr${}_{0.9}$Sb${}_{0.1}$O${}_{6}$ (100%)	8.356 (1)	Ba	1	0.25	0.25	0.25	0.357 (4)
			Y	1	0.00	0.00	0.00	0.276 (1)
			Ir	0.90	0.50	0.50	0.50	0.225 (3)
			Sb	0.10 (1)	0.50	0.50	0.50	0.225
			O	1	0.263 (5)	0.00	0.00	0.205 (2)
BYISO-20	Ba${}_{2}$YIr${}_{0.78}$Sb${}_{0.22}$O${}_{6}$ (91%)	8.364 (1)	Ba	1	0.25	0.25	0.25	0.388 (2)
			Y	1	0.00	0.00	0.00	0.306 (2)
			Ir	0.78	0.50	0.50	0.50	0.295 (1)
			Sb	0.22 (2)	0.50	0.50	0.50	0.295
			O	1	0.262 (9)	0.00	0.00	0.191 (4)
	Ba${}_{2}$YIrO${}_{6}$ (9(1)%)	8.357 (1)	Ba	1	0.25	0.25	0.25	0.188
			Y	1	0.00	0.00	0.00	0.316
			Ir	1	0.50	0.50	0.50	0.195
			O	1	0.262 (1)	0.00	0.00	0.191
BYISO-50	Ba${}_{2}$YIr${}_{0.41}$Sb${}_{0.59}$O${}_{6}$ (85%)	8.384 (1)	Ba	1	0.25	0.25	0.25	0.363 (1)
			Y	1	0.00	0.00	0.00	0.288 (3)
			Ir	0.41	0.50	0.50	0.50	0.257 (3)
			Sb	0.59 (3)	0.50	0.50	0.50	0.257
			O	1	0.262 (2)	0.00	0.00	0.211 (5)
	Ba${}_{2}$YIrO${}_{6}$ (15(1)%)	8.359 (1)	Ba	1	0.25	0.25	0.25	0.363
			Y	1	0.00	0.00	0.00	0.288
			Ir	1	0.50	0.50	0.50	0.257
			O	1	0.262 (3)	0.00	0.00	0.211
BYISO-80	Ba${}_{2}$YIr${}_{0.12}$Sb${}_{0.88}$O${}_{6}$ (91%)	8.408 (1)	Ba	1	0.25	0.25	0.25	0.284 (9)
			Y	1	0.00	0.00	0.00	0.369 (7)
			Ir	0.12	0.50	0.50	0.50	0.378 (8)
			Sb	0.88 (1)	0.50	0.50	0.50	0.378
			O	1	0.263 (3)	0.00	0.00	0.378 (8)
	Ba${}_{2}$YIrO${}_{6}$ (9(1)%)	8.356 (1)	Ba	1	0.25	0.25	0.25	0.284
			Y	1	0.00	0.00	0.00	0.369
			Ir	1	0.50	0.50	0.50	0.378
			O	1	0.267 (1)	0.00	0.00	0.378

**Table 2:** Observed distances for Ir-O, Sb-O, and Y-O from XRD analysis of phase I in BYIO, BYISO-10, BYISO-20, BYISO-50, BYISO-80, and BYSO [16,19,44].

Compounds	Bond Length in Å	
	Ir(Sb)-O	Y-O
BYIO	1.98	2.19
BYISO-10	1.97	2.20
BYISO-20	1.98	2.20
BYISO-50	1.99	2.19
BYISO-80	1.99	2.21
BYSO	1.96	2.24

**Table 3:** Local structure parameters as obtained from the EXAFS analysis of the Ir $L_{3}$ edge for the four samples. The absolute mismatches between the experimental data and the best fit are $R^{2}$ = 0.038, 0.042, 0.047, and 0.05 for BYISO-10, BYISO-20, BYISO-50, and BYISO-80, respectively. Uncertainty on the last digit of the refined parameters is reported in parentheses. The values of fixed or constrained parameters have no uncertainties.

Sample	Shell	N	$\sigma^{2}$ × $10^{-3}$ (Å${}^{2}$)	*R* (Å)
BYISO-10	Ir-O	6	0.18 (2)	1.961 (6)
	Ir-O-O	24	0.9 (1)	3.31 (3)
	Ir-Ba	8	13 (2)	3.62 (3)
	Ir-Y (SS)	6	1.1 (1)	4.17 (3)
	Ir-O-Y (MS-3 legs)	12	6.6	4.17
	Ir-O-Y-O (MS-4 legs)	6	12.1 (1)	4.17
BYISO-20	Ir-O	6	0.27 (3)	1.968 (6)
	Ir-O-O	24	1.1 (2)	3.36 (3)
	Ir-Ba	8	14 (2)	3.64 (2)
	Ir-Y (SS)	6	2.7 (2)	4.20 (2)
	Ir-O-Y (MS-3 legs)	12	5.8	4.20
	Ir-O-Y-O (MS-4 legs)	6	8.8 (4)	4.20
BYISO-50	Ir-O	6	0.15 (2)	1.962 (6)
	Ir-O-O	24	1.2 (2)	3.31 (3)
	Ir-Ba	8	12 (1)	3.63 (2)
	Ir-Y (SS)	6	2.7 (2)	4.21 (2)
	Ir-O-Y (MS-3 legs)	12	5.0	4.21
	Ir-O-Y-O (MS-4 legs)	6	7.4 (3)	4.21
BYISO-80	Ir-O	6	0.13 (1)	1.958 (6)
	Ir-O-O	24	2.0 (2)	3.25 (3)
	Ir-Ba	8	10 (1)	3.53 (2)
	Ir-Y (SS)	5.5 (1)	3.8 (2)	4.24 (2)
	Ir-O-Y (MS-3 legs)	11	4.5	4.24
	Ir-O-Y-O (MS-4 legs)	5.5	5.2 (2)	4.24
	Ir-Ir (SS) (ASD)	0.5	9.5 (2)	4.24
	Ir-O-Ir (MS-3 legs)	1.0	7.8	4.24
	Ir-O-Ir-O (MS-4 legs)	1.0	5.3 (2)	4.24

**Table 4:** X-ray photoelectron spectroscopy (XPS) data for the binding energy (eV) of $f_{7/2}$ orbital and spin–orbit separation of Ir${}^{5+}$ element.

Sample	Energy (eV)	SO Splitting (eV)
BYISO-10	63.18	3.05
BYISO-20	63.21	3.04
BYISO-50	63.15	3.04
BYISO-80	63.13	3.04

**Table 5:** Effective paramagnetic moments from CW fitting and phase I moments calculated from XRD phase percentage (considering phase II moment ∼0.44 $\mu_{B}$/Ir) of BYIO [17], BYISO-10, BYISO-20, BYISO-50, and BYISO-80 [16].

	Overall Compound		Phase I	
Sample	$\Theta_{\mathrm{CW}}$ (K)	$\mu_{\mathrm{eff}}$ ($\mu_{B}$/Ir)	Sb (%)	$\mu_{\mathrm{Phase}I}$ ($\mu_{B}$/Ir)
BYISO-10	−96	0.45	10	0.45
BYISO-20	−76	0.47	22	0.47
BYISO-50	−44	0.57	59	0.59
BYISO-80	−31	0.67	88	0.69

## Data Availability

XAFS data will be available at the ELETTRA-XAFS beamline after an embargo (see https://www.elettra.eu/userarea/scientific-data-policy.html (accessed on 1 January 2024) for the ELETTRA synchrotron data policy).
